# Efficacy and Mechanism of Cinnamon Essential Oil on Inhibition of *Colletotrichum acutatum* Isolated From ‘Hongyang’ Kiwifruit

**DOI:** 10.3389/fmicb.2018.01288

**Published:** 2018-06-18

**Authors:** Jingliu He, Dingtao Wu, Qing Zhang, Hong Chen, Hongyi Li, Qiaohong Han, Xingyue Lai, Hong Wang, Yingxue Wu, Jiagen Yuan, Hongming Dong, Wen Qin

**Affiliations:** ^1^Sichuan Key Laboratory of Fruit and Vegetable Postharvest Physiology, College of Food Science, Sichuan Agricultural University, Ya’an, China; ^2^Faculty of Agricultural, Life & Environmental Sciences, University of Alberta, Edmonton, AB, Canada

**Keywords:** kiwifruit, *Colletotrichum acutatum*, pathogens, cinnamon essential oil, antifungal mechanism

## Abstract

In this study, one of the dominant pathogens, which caused postharvest diseases such as anthracnose, was isolated from decayed ‘Hongyang’ kiwifruit. It was identified as *Colletotrichum acutatum* by its morphological characteristics and standard internal transcribed spacer ribosomal DNA sequence. Further, the efficacy and possible mechanism of cinnamon essential oil on inhibition of *C. acutatum* were investigated. Results showed that *C. acutatum* was dose-dependently inhibited by cinnamon essential oil. Meanwhile, the mycelial growth and spore germination of *C. acutatum* were completely inhibited at the concentrations of 0.200 μL/mL and 0.175 μL/mL (*v/v*), respectively. Indeed, both minimal inhibitory and minimum fungicidal concentrations of cinnamon essential oil were measured as 0.200 μL/mL. Additionally, the possible antifungal mechanism of cinnamon essential oil on *C. acutatum* was demonstrated. Results showed that the cinnamon essential oil could destroy the cell membrane integrity of *C. acutatum*, and the structure of cell membrane was changed. Indeed, the cell cytoplasm including soluble protein, sugar, and nucleic acid was released, which significantly changed the extracellular conductivity. Results suggested that the cinnamon essential oil exerted great potential to be used as a natural and efficient preservative for kiwifruit postharvest storage, which were helpful for the better understanding of the efficacy and mechanism of cinnamon essential oil on inhibition of pathogens isolated from decayed ‘Hongyang’ kiwifruit.

## Introduction

‘Hongyang’ kiwifruit (*Actinidia chinensis*) is the first international red-fleshed cultivar in Sichuan of China. Due to its unique flavor and abundant nutrients, such as high levels of vitamin C, anthocyanins, dietary fiber, and amino acids, ‘Hongyang’ kiwifruit has interested consumers (Lin et al., 2017). China is the leading kiwifruit producing country, and the average level of production topped the list in 2013–2016 (44.5% of the world production). Kiwifruit is infected by different fungi associated with fruit diseases. For instance, gray mold, caused by *Botrytis cinerea*, is one the important postharvest diseases of kiwifruit (Williamson et al., 2007); Blue mold, caused by *Penicillium expansum*, can also cause decay in harvested kiwifruit, although it is not as prevalent as gray mold (Neri et al., 2010). Especially, the anthracnose of kiwifruit is severe, which can infect leaves, canes, and fruits, resulting in significantly economic losses during storage. However, at present, relevant studies on anthracnose of kiwifruit have seldom been reported.

The use of chemically synthetic preservative in controlling food spoilage and pathogenic fungus has been a controversial topic (Tian et al., 2014). The artificially chemical compounds are considered as chronic and reproductive toxicants causing respiratory diseases or other health risks (Fleming-Jones and Smith, 2003). In this case, natural preservatives such as essential oil (EO) have been extensively used due to its biodegradable and antimicrobial properties. EO contains a variety of substances called ‘phytochemicals’, which belong to natural components in plants (Pratt, 1992). The phytochemical preparations with dual functionalities in preventing lipid oxidation and antimicrobial properties have tremendous potential for extending shelf life of food products (Singh et al., 2007; Fadli et al., 2012). Generally, EO possesses high volatility. When EO is applied as a vapor, less oil is used. Further, the residues of EO on the product are minimized, and there will be less of a problem with tainting (Szczerbanik et al., 2007). It may be more appropriate to use EO in their vapor phase for postharvest applications (Concha et al., 1998; Zollo et al., 1998). Cinnamon (*Cinnamomum zeylanicum* or *Cinnamomum verum*), rich in EO, belongs to Lauraceae family comprising about 250 species and usually distributes in India, China, Sri Lanka, and Australia (Prasad et al., 2009). Cinnamon essential oil (CEO) is a promising food preservative for the inhibition of foodborne pathogens and spoilage microorganisms (Tian et al., 2012). Our previous studies have shown that CEO have good efficacy on preservation of ‘Hongyang’ kiwifruit. Moreover, ‘Hongyang’ kiwifruit fumigated with 0.4 μL/mL of CEO could be stored at (4 ± 1) °C with relative humidity of 90–95% for 120 days (He et al., 2015). CEO has been demonstrated as a strong and broad range of inhibition for bacteria, fungi and yeast (Chao et al., 2000; Manso et al., 2015). Our previous studies have demonstrated that CEO have good inhibitory effects against *Botryosphaeria parva in vitro*, and can reduce soft rot on ‘Hongyang’ kiwifruit *in vivo* (He et al., 2015). Antifungal activity of CEO against *C. acutatum* isolated from strawberry anthracnose was investigated (Duduk et al., 2015). Cell plasma membrane (PM) is the action site invaded by antifungal substances, such as silicon and chlorine dioxide (Wei et al., 2008; Liu et al., 2010). For fungi, the integrity of PM plays a crucial role in maintaining cell constituents being important to viability, such as sugar, protein and nucleic acid. The soluble sugar, soluble protein and nucleic acid are the basic and functional components in the cell. The PM is damaged, and the intracellular components can be leaked (Wei et al., 2008; Kim et al., 2009; Liu et al., 2010). The antifungal activity of EO was strongly associated with its compositions, such as monoterpenic phenoles, especially thymol, carvacrol, and eugenol (Barrera-Necha et al., 2008). The antifungal mechanism of EO is speculated to induce membrane disruption by their lipophilic compounds (Cowan, 1999). The low-molecular-weight and highly lipophilic components of EO pass easily through cell membranes and cause disruption to the fungal cell organization (Chao et al., 2005; Shukla et al., 2012). Therefore, in this study, the dominant pathogens, which caused postharvest diseases such as anthracnose in ‘Hongyang’ kiwifruit, was firstly isolated and identified. Further, the antifungal activity and the possible antifungal mechanism of CEO against *C. acutatum* isolated from decayed ‘Hongyang’ kiwifruit were investigated.

## Materials and Methods

### Materials and Chemicals

Fresh ‘Hongyang’ kiwifruits grown in Ya’an country, Sichuan Province, China, were harvested from orchards. The spoiled kiwifruits were utilized for the isolation of pathogens in Sichuan Agricultural University of China. Potato dextrose agar (PDA) (Beijing AoBoXing bio-tech Co. Ltd., Beijing, China) was used as the culture media. The fungal DNA was extracted using a commercial kit (Eppendorf, Holstein City, Germany), following the manufacturer’s instructions. Whatman No. 1 filter paper disks (Solarbio, Shanghai, China) were cut (Ø = 5 mm) using a hole-puncher. The crude cinnamon essential oil (CEO, Ji’an Guoguang flavor Co. Ltd., Jiangxi, China) was obtained by hydrodistillation from cinnamon bark, and the composition of the CEO used in this study was given in **Table 1**.

**Table 1:** Chemical compositions of CEO.

No.	Components	RT (S)	RI	PA (%)
1	Benzaldehyde	6.998	750	2.68 ± 0.04
2	*trans*-Cinnamaldehyde	15.945	1567	86.16 ± 0.10
3	*trans*-Cinnamic acid	20.829	1093	6.75 ± 0.12
4	Others non-identified			<5.00

*Phytochemical analysis was conducted by using GC-MS, and data were the mean (±SD) of three independent analysis. RT, Retention time; RI, Linear retention index to n-alkane C8 - C40 on silica capillary column; % PA, Percentages of the relative areas (%), the data were obtained from the integration of the peaks identified in the spectra by a selective mass detector.*

### Isolation and Identification of Pathogens

According to the tissue separation method (Jogee et al., 2017), the surface of the spoiled ‘Hongyang’ kiwifruits was washed with sterile double-distilled water (SDW), and disinfested in 75% ethanol for 30 s and in 1% sodium hypochlorite for 30 s, and then rinsed three times in SDW. Then, pieces of kiwifruit sarcocarp about 4 mm^2^ were excised from diseased berries with a sterile scalpel from the marginal area between the diseased and healthy tissue. The pieces were incubated on PDA. After isolation, the purified fungi were cultivated on PDA. After incubation at 25°C for 7 days, colonial morphology including micromorphological features, viz., color of colony on both of dorsal and ventral sides, growth diameter and texture of colony, and some microscopic features like mycelial size and conidial shape were measured. The isolated pathogen was assayed its pathogenicity in ‘Hongyang’ kiwifruit. Further, the pathogen was isolated again from diseased kiwifruits using the above tissue separation method. Finally, the pathogen was subcultivated on PDA and stored at 4°C for consequent identification. The isolated pathogen was coded as WQ1. The pathogen was deposited in Agricultural Culture Collection of China, and the accession number was ACCC 39342.

The isolate was grown on potato dextrose broth (PDB) with 140 r/min at 25°C for 5 days. The genomic DNA of mycelia was extracted by kit (Eppendorf, Holstein City, Germany). The internal transcribed spacer (ITS) regions and the small subunit (ITS1-5.8S-ITS2) of the rDNA genes were amplified using the primer set ITS1 (5′-TCCGTAGGTGAACCTGCGG-3′) and ITS4 (5′-TCCTCCGCTTATTGATATGC-3′) (Zhao et al., 2010). Primers were synthesized by Shanghai Sangon Biological Engineering Technology & Services Co., Ltd (Shanghai, China). PCR amplification was carried out in aqueous volumes of 25 μL. The reactions contained 0.5 μL template DNA (30 ng), 0.5 μL (10 μmol/L) of each primer, 5 × PCR buffer (with MgCl_2_) 2.5 μL, 0.5 μL dNTP (2.5 mmol/L), 0.5 μL Taq polymerase (2.5 U/μL) and 20 μL ddH_2_O. PCR reactions were performed on a ABI 2720 thermal cycler (Applied Biosystems, Foster City, CA, United States). Thermal cycling was carried out using an initial denaturation step at 98°C for 3 min, followed by 30 cycles of denaturation at 98°C for 25 s, annealing at 55°C for 25 s and extension at 72°C for 60 s. Cycling was completed by a final elongation step at 72°C for 10 min. PCR products were purified using PCR purification kit (Shanghai Sangon Biological Engineering Technology & Services Co., Ltd, Shanghai, China). Sequencing was performed with an ABI 3730XL automated sequencer (Applied Biosystems, Foster City, CA, United States). Further, the sequence was analyzed and determined to search for similar sequences from the Basic Local Alignment Search Tool (BLAST) software^1^ algorithm at National Center for Biotechnology Information (NCBI). To construct the relevant phylogenic tree, MEGA 5.02 software was employed (Saitou and Nei, 1987; Tamura et al., 2007).

### Analysis of Antifungal Activity of Cinnamon Essential Oil Against Mycelial Growth and Spore Germination

The inhibitory effect of CEO on mycelial growth was determined using filter paper method with minor modifications (Liu et al., 2010). Briefly, the mycelia disks (Ø = 5 mm) of *C. acutatum*, cut from the edge of 7 days actively growing cultures on PDA, were placed upside down on the center of the inner side of the plate lid with 20 mL of PDA. The sterile filter paper (Ø = 5 mm) was added with different amounts of CEO (4.5, 6.0, 7.5, 9.0, 10.5, 12.0, and 13.5 μL). The filter paper containing CEO were placed on the center of the bottom of the petri dishes to obtain final concentrations of 0.075, 0.100, 0.125, 0.150, 0.175, 0.200, and 0.225 μL/mL of air (*v/v*). PDA plates without CEO were used as negative controls. Each plate was sealed with Parafilm^®^ (Top Group Co. Ltd., Chengdu, China) to prevent leakage of CEO vapor and incubated in the dark at 25°C until the growth in the control plates (without treatment) reached the edge of the plates. The plates were incubated in inverted position. Each treatment was replicated thrice and the experiment was repeated thrice. The inhibition radius around the oil disk (colony-free perimeter) was measured using a digital vernier caliper (Mitutoyo, Kawasaki, Japan). Mycelia growth (cm) was recorded and the percentage inhibition (PI) was determined after comparison with the control (Pandey et al., 1982; Trabelsi et al., 2016). The minimal inhibitory concentration (MIC) was defined as the lowest concentration of CEO at which the growth of microorganism was inhibited (Rasooli and Abyaneh, 2004). The fungus treated with the MIC of CEO was sub-cultivated on treatment-free PDA at 25°C for 2 days to determine whether the inhibition was reversible. The minimal fungicidal concentration (MFC) was regarded as the lowest concentration in which the growth of any fungal colony was prevented on PDA (Irobi and Daramola, 1993).

PI(%)=(dc−dt)/dc×100

where dt is the average diameter of colony after treatment by CEO, and dc is the average diameter of colony used for control.

The inhibitory effect of CEO on spore germination and germ tube elongation of *C. acutatum* was determined using a vapor contact method proposed from previous study with minor modifications (Liu et al., 2007). To stimulate sporulation, *C. acutatum* was grown on PDA in the dark at 25°C. Spores were harvested from 7 days old cultures with 10 mL of SDW and softly scraping the colonies with a sterile L-shaped glass rod. Spore suspension was filtered through sterile paper to remove mycelial fragments. Hundred microlliter of spore suspension of 10^8^ CFU/mL was plated on the inner side of petri dishes (Ø = 90 mm) with 20 mL of PDA. The same CEO concentrations used in the mycelial growth assay were examined. Each plate was incubated in the dark at 25°C for 1 days. In the end, germinal spores were observed using a light microscope (Olympus CX 31, Olympus Co., Tokyo, Japan) at 400× magnification. Each slide was fixed in lactophenol cotton blue. Results were expressed in terms of the percentage of spore germination inhibition by comparing control and treated plates (Trabelsi et al., 2016; Ribes et al., 2017).

Spore germination inhibition (%)=(sc−st)/sc×100

where sc and st are the average numbers of spores germinated in control plates and treated plates, respectively.

### Observations of Fungal Morphology and Ultrastructure

The action mechanism of CEO was determined using transmission electron microscopy (TEM) according to a previous study with minor modifications (Bozzola and Russell, 1999). Seven-day-old *C. acutatum* exposed to 0, MFC and 2MFC of CEO were cultivated in the dark at 25°C for 2 days. The mycelia was harvested from PDA with 10 mL of SDW and softly scraping the colonies with a sterile L-shaped glass rod. The mycelium suspension was centrifuged at 7000 *g* for 15 min to obtain mycelia. The small segments measuring 5 × 5 mm were excised at the margin of the mycelium colony. Then, the segments were promptly placed in vials containing 2.5% glutaraldehyde in 0.1 mol/L phosphate buffer saline (PBS) (pH 7.2) at 4°C and fixed overnight. The fixed samples were rinsed with the same buffer three times for 10 min. Afterward, the samples were dehydrated in a graded series of ethanol (70, 80, 90, 95, and 100%, *v/v*) for 20 min in each alcohol dilution. The last step was performed for 30 min thrice. The dehydrated specimens were then embedded and polymerized in Spurr’s resin at 65°C for 72 h. Ultrathin sections (approximately 50 nm in thickness) were hand trimmed with a diamond knife using an MT-X Ultratome for TEM observations (H-600, Hitachi Co. Ltd., Tokyo, Japan).

Membrane integrity was determined using fluorescent microscope (FSM) according to a previous study with minor modifications (Liu et al., 2010). *C. acutatum* was treated with the MFC of CEO as described previously. PDA plates without CEO were served as negative controls. Mycelia were collected after incubation for 0, 1, 2, 3, 4, 5, and 6 days in the dark at 25°C, and washed with cold PBS (0.05 mol/L, pH 7.0) to remove residual medium, respectively. Mycelia were then fixed by cold ethanol (70%, *v/v*) at 4°C for 1 h. After removal of ethanol, and the mycelia were washed twice with PBS. Then the mycelia were stained with 50 μg/mL propidium iodide for 20 min at 4°C in the dark. Mycelia were collected by centrifugation (4000 ×*g* for 10 min), and washed twice with PBS to remove residual dye. Samples were observed with the E200 Nikon Eclipse microscope (Nikon Co., Tokyo, Japan). Fields of view from each cover slip were chosen randomly, and each treatment was replicated thrice and the experiment was repeated thrice. The number of spores in bright-field was defined as the total number, and membrane integrity (MI) was calculated as:

$$\text{MI}(\%)=\left[1-\left(\frac{\text{number of stained spores}}{\text{number of total spores}}\right)\right]\times 100$$

### Effects of Cinnamon Essential Oil on Ergosterol Content in Plasma Membrane

The ergosterol content was determined using a previously described method with minor modifications (Tian et al., 2012). The same treatment used in the mycelial growth assay was examined. After incubation, mycelia were harvested and washed with 10 mL of SDW. A 5 mL of mixed solution (20 mL of methanol, 5 mL of ethanol and 2.0 g of potassium hydroxide) was added to each sample. After 2 min of vortex using TS-1 Eddy oscillometer (Kylin-Bell Lab Instruments Co., Ltd., Shanghai, China), the mixed solution was incubated at 85°C for 4 h. Sterols were extracted from each sample by adding a mixture of 2 mL of SDW and 5 mL of *n*-heptane. Then, the mixture was sufficiently mixed by vortex for 2 min allowing the layers to separate for 1 h at room temperature. The *n*-heptane layer was analyzed by UV-1800 PC spectrophotometer at 230 and 282 nm (Shanghai AoXi Science Instrument Co. Ltd, Shanghai, China). The presence of ergosterol (at 282 nm) and the sterol intermediate 24(28) dehydroergosterol (at 230 nm) in the *n*-heptane layer led to a characteristic curve. The content of ergosterol was calculated as a percentage of the wet weight based on the absorbance and wet weight of the initial pellet. It was calculated as the follow equation,

$$\text{\% ergosterol}=\frac{\frac{\text{A282}}{290}}{\text{wet weight}}-\frac{\frac{\text{A230}}{518}}{\text{wet weight}}$$

where 290 and 518 are the *E* values (in percentages per cm) determined for crystalline ergosterol and 24(28) dehydroergosterol, respectively.

### Effects of Cinnamon Essential Oil on Intracellular Protein, Sugar, and Nucleic Acid Leakage

The effects of CEO on intracellular leakage were determined using a extracellular conductivity method with minor modifications (Tao et al., 2014). Initially, the mycelia disks (Ø = 5 mm) were placed upside down on the center of Petri dishes with 20 mL of PDA and incubated in the dark at 25°C until the growth reached the edge of the plates. Then, the sterile filter paper was added with the MFC of CEO, and PDA plates without CEO were served as negative controls. Mycelia were collected after incubation for 0, 1, 2, 3, 4, 5, and 6 days in the dark at 25°C, and washed with 15 mL of SDW. The supernatants were collected after centrifugation. The extracellular conductivity at each time point was carried out using a DDS-11A conductivity meter (Shanghai Precision Scientific Instrument Co. Ltd, Shanghai, China) and expressed as μS/cm. Each treatment was replicated thrice and the experiment was repeated thrice.

The intracellular protein, sugar and nucleic acid leakage of mycelia were determined by a spectrophotometry method with minor modification (Fleissner and Glass, 2007). Briefly, *C. acutatum* was treated with the MFC of CEO as described above. PDA plates without CEO were served as negative controls. Mycelia were collected after incubation for 0, 1, 2, 3, 4, 5, and 6 days, and washed with 15 mL of SDW. The mycelium suspension was centrifuged at 4000 ×*g* for 10 min to obtain mycelium, and the filtrate was collected for the determination of the leakage of intracellular content by the assays of total soluble protein, total soluble sugar, and nucleic acid. The content of soluble protein was determined with bovine serum albumin (Solarbio, Shanghai, China) as standard (Bradford, 1976). The content of soluble sugar was estimated by the phenol-sulfuric acid method using glucose (Solarbio, Shanghai, China) as standard (Dubois et al., 1956). The protein or sugar leakage was expressed as g/kg wet weight of mycelia. To determine the concentration of the released nucleic acid, 1 mL of supernatant was used to measure the absorbance at 260 nm with a UV-1800 PC spectrophotometer (Shanghai AoXi Science Instrument Co. Ltd, Shanghai, China) (Zhang et al., 2016). Each treatment was replicated thrice and the experiment was repeated thrice.

### Statistical Analysis

Results were expressed as means ± standard deviation (SD) of three independent repeated experiments, as the interaction between treatment and experiment variables was not significant. Statistically significant differences between mean values were analyzed with one-way analysis of variance (ANOVA) and Duncan’s multiple range tests using SPSS 19.0 (IBM, New York, NY, United States). Differences at *p* < 0.05 were considered as statistically significant.

## Results

### Isolation and Identification of *C. acutatum*

**FIGURE 1:**
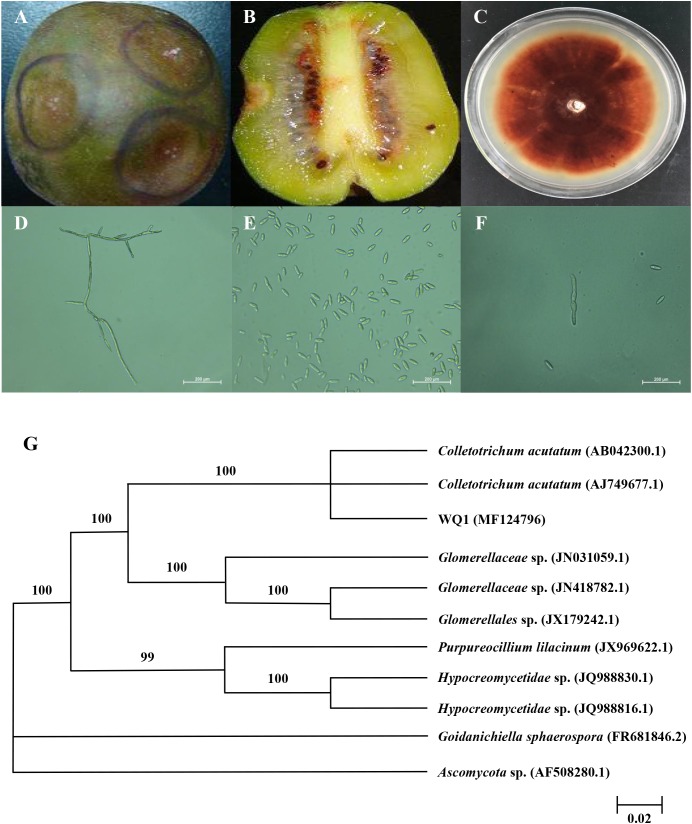
Symptoms of kiwifruit anthracnose **(A,B)**, and cultural **(C)** and morphological **(D–F)** characteristics of isolated pathogen, as well as its phylogenetic tree **(G). (A)** Symptoms on the surface of diseased kiwifruit; **(B)** Symptoms inside of diseased kiwifruit; **(C)** Colonies of pathogen cultured for 7 days at 25°C; **(D)** Mycelia of pathogen and 400× magnification; **(E)** Spores of pathogen and 400× magnification; **(F)** Germinal spores and 400× magnification; **(G)** Phylogenetic analysis of DNA sequences obtained from fragments of the ITS rDNA from the isolate along with the reference sequences from NCBI. The analysis was conducted using neighbor joining method. The scale bar represents 0.02% substitutions of nucleotide.

The kiwifruit infected by anthracnose appeared on round spots from brown to dark brown launched from center, whose surface was depression, dried-up, and severe dehydration, while the tissue obviously turned into soft (**Figure 1A**). The pathogenic sarcocarp turned from green into pale yellow. Subsequently, the whole fruit was rotten (**Figure 1B**). The colony of pathogen was circular on PDA after 7-day incubation, and its texture was soft and villous, as well as the color was pink (**Figure 1C**). The aerial mycelia grew radially from the center to the surrounding area. Mycelia were hyaline and septate (**Figure 1D**). The ellipsoidal spores produced the pale pink spore heaps, while the spore was single, colorless, and hyaline, diameter of 13.1–18.6 × 3.0–4.0 μm (**Figure 1E**). The germinal spore was spheroidal and the diameter of the germinal spore was 11.0–15.0 × 2.0–3.0 μm (**Figure 1F**). Thus it could be considered as *Colletotrichum* sp. It was verified that the isolated pathogen could cause the kiwifruit anthracnose through pathogenicity test. The colonial morphology and some microscopic features of the isolated pathogen were accordance with previous observations.

To further identify the isolated pathogen, the ITS1-5.8S-ITS2 region of isolate was sequenced. The PCR product was 531 bp. The obtained sequence was submitted to GenBank, and the accession number was MF 124796. The ITS sequence was preliminarily analyzed and submitted to GenBank as closest to those of *Colletotrichum* sp. For the identification purposes, the sequence was compared to those available in the NCBI database using the BLAST. Furthermore, the homology sequences were analyzed with MEGA 5.02 software to construct phylogenetic tree by the neighbor-joining method (**Figure 1G**). Confidence values above 50% obtained from a 1,000-replicate bootstrap analysis were shown at the branch nodes. Bootstrap values from neighbor-joining method were determined. *Goidanichiella sphaerospora* (FR 681846.2) and *Ascomycota* sp. (AF 508280.1) were used as the out group. The isolated pathogen had higher similar sequences of *C. acutatum* than any other reference taxa. In the neighbor-joining tree, the anthrax pathogen and other three reference taxa including *Glomerellaceae* sp., *Purpureocillium lilacinum* and *Hypocreomycetidae* sp. formed a clade with 100% bootstrap support. In this clade, the reference taxon, *C. acutatum* (AB 042300.1) and *C. acutatum* (AJ 749677.1) also clustered together with it, meanwhile they were same species. The result of similarity comparisons of the ITS1-5.8S-ITS2 region sequence revealed that isolated pathogen had the highest nucleotide similarities with *C. acutatum*.

### Effect of CEO on *C. acutatum in Vitro*

**FIGURE 2:**
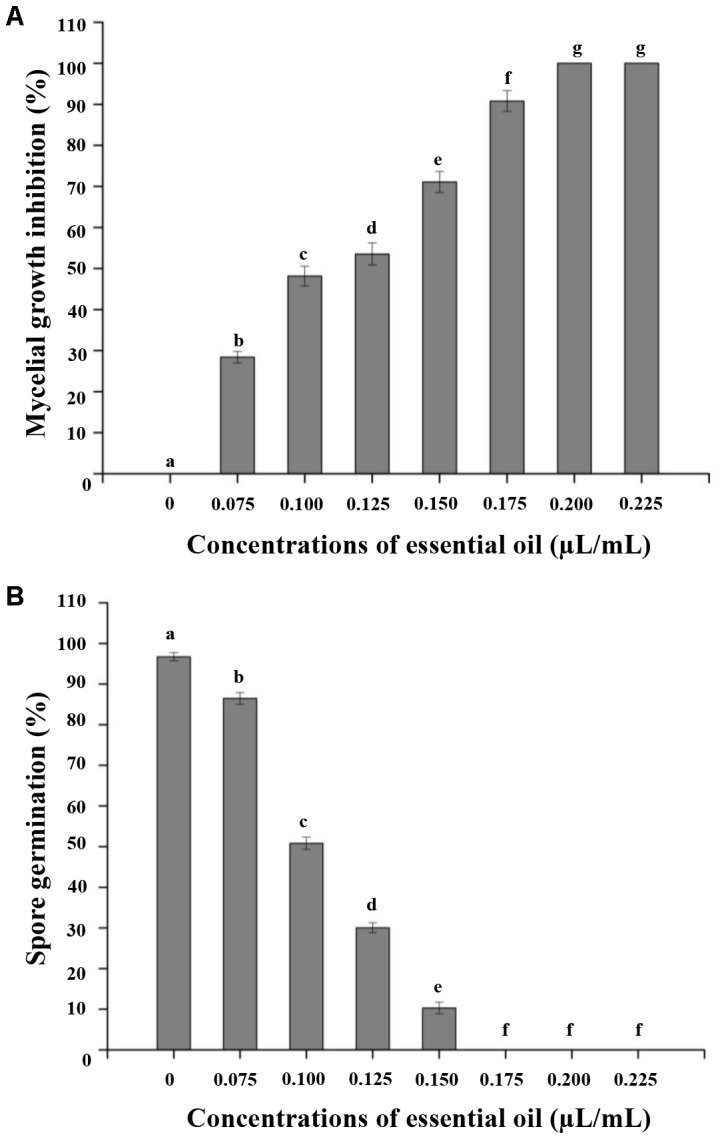
Effect of different concentrations of CEO on mycelial growth **(A)** and spore germination **(B)** of *C. acutatum.* Values are the averages of the replicates for all the analyses. Error bars are ±SD of the means. In some cases the error bar is obscured by the symbol. Columns with different letters at each time point indicate significant differences according to Duncan’s multiple range tests at *p* < 0.05.

We evaluated the antifungal activity of CEO *in vitro* against *C. acutatum.* The antifungal activity was mainly determined by inhibition of mycelial growth and spore germination of *C. acutatum*. The mycelial growth of *C. acutatum* was sensitive to CEO (**Figure 2A**). The mycelial growth of *C. acutatum* (CEO-treated group) was reduced during incubation compared with the untreated group, with the greater inhibitory at the higher concentration (*p* < 0.05). The mycelial growth of *C. acutatum* was completely inhibited by CEO at the concentration of 0.2 μL/mL. The efficacies of CEO on the spore germination of *C. acutatum* were shown in **Figure 2B**. The different concentrations of CEO had a significant inhibitory effect on spore germination (**Figure 2B**, *p* < 0.05). Observations showed an inhibition on the spore germination of *C. acutatum* within the range of 0.075–0.150 μL/mL. Results indicated that the spore germination was reduced with the increasing CEO concentrations. CEO could completely inhibit the spore germination at the concentration of 0.175 μL/mL. In summary, the CEO completely prevented the mycelial growth and spore germination of *C. acutatum* at concentrations of 0.2 μL/mL and 0.175 μL/mL, respectively. Further, the MIC and MFC values of CEO against *C. acutatum* were presented in **Table 2**. The MIC of CEO was 0.200 μL/mL. The MFC of CEO was found to be equal to the corresponding MIC results. CEO showed the good fungistatic and fungicidal activity against *C. acutatum.*

**Table 2:** MIC and MFC of CEO against *C. acutatum.*

Concentration of CEO (μL/mL)	MIC	MFC
0	++++	++++
0.075	+++	+++
0.100	+++	+++
0.125	++	++
0.150	++	++
0.175	+	+
0.200	–	–
0.225	–	–

*Growth of C. acutatum in the presence of CEO. Medium used: PDA. Inoculating in dark at 25°C. Growth: ++++, very good; +++, good; ++, fair; +, little; -, no growth. Values are mean (n = 3).*

### Effects of CEO on Morphology and Ultrastructure of *C. acutatum*

**FIGURE 3:**
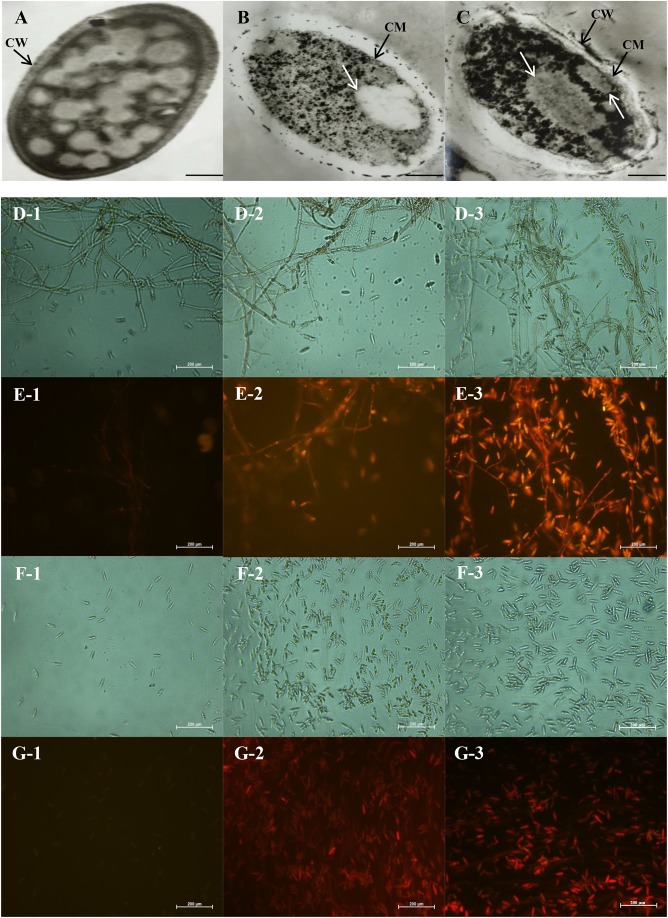
Transmission electron microscopy **(A–C)** and fluorescence microscopy images **(D–G)** of antifungal effect of CEO against *C. acutatum*. **(A)** Healthy mycelia with control, the magnitudes of 15000×; **(B)** Mycelia treated with the MFC of CEO; **(C)** Mycelia treated with the 2MFC of CEO; **(D1–D3)** Bright field of mycelia in microscopy after 0, 3, and 6 days of incubation, in the magnitudes of 400×; **(E1–E3)** Propidium iodide of mycelia in microscopy after 0, 3, and 6 days of incubation; **(F1–F3)** Bright field of spores in microscopy after 0, 3, and 6 days of incubation; **(G1–G3)** Propidium iodide of spores in microscopy after 0, 3, and 6 days of incubation.

Transmission electron microscopy could intuitively reflect the morphological alterations of the cell wall (CW), cell membrane (CM) and cytoplasm. The growth inhibition of *C. acutatum* induced with different concentrations of CEO for 2 days was found to be well correlated with morphological changes of fungi exposed to control, MFC and 2MFC of CEO. The morphological changes of untreated and treated fungal cell were shown in **Figure 3**. In the control samples, the cells were dense appearance (**Figure 3A**). Results showed that the CW was uniform and thoroughly surrounded by an intact fibrillar layer for untreated fungi. Indeed, the CM was unfolded and uniform in shape (**Figure 3A**). All organelles had a normal appearance, and were clearly observed. In treated fungi, the destroyed cell structures were marked in CW and organelles (**Figures 3B,C**). The major disruption was the endomembrane system, containing the CM and membranous organelles. *C. acutatum* was treated with the MFC of CEO, the CW was deformed (**Figure 3B**). The fibrillar layers gradually lost their constitutions, becoming thinner and eventually detaching from the CW (**Figure 3B**). The fibrillar layers were hardly observed when the cells were treated with CEO of 2MFC (**Figure 3C**). The CM of *C. acutatum* treated with CEO lost its linear structure, becoming rough and villous with invaginations of vesicles. The CM was ruptured and detached from the cytoplasm (**Figures 3B,C**). After treatment with CEO, most organelles were indistinct and many structures were unidentifiable (**Figures 3B,C**). The intracellular organization was noticeably disrupted with uneven distribution, showing cytoplasmic condensation and absent (**Figures 3B,C**). Moreover, the damage in the intracellular organization was more severe with the increasing concentration of CEO (**Figures 3B,C**).

**FIGURE 4:**
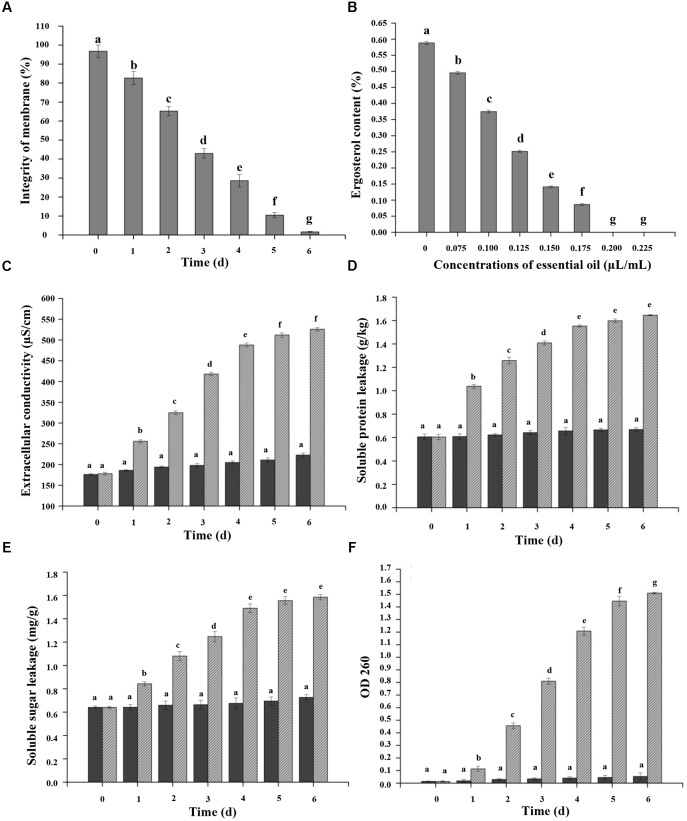
Effects of CEO on percentage of plasma membrane integrity **(A)**, ergosterol content **(B)**, extracellular conductivity **(C)**, and protein **(D)**, sugar **(E)**, and nucleic acid **(F)** leakage of *C. acutatum*. **(A)** Percentage of plasma membrane integrity of *C. acutatum* spores, *C. acutatum* was cultured in PDA containing CEO or in PDA without CEO as the control at 25°C; **(B)** Ergosterol contents of *C. acutatum* on PDA containing different concentrations of CEO at 25°C were assayed; **(C)** Cellular leakage from fungal tissues was determined 0–6 days after incubation with the CEO. Mycelia were cultured in PDA containing CEO or in SDW without EO as the control at 25°C. Samples for the leakage were measured for 6 days; **(D)** Soluble protein leakage of *C. acutatum*; **(E)** Soluble sugar leakage of *C. acutatum*; **(F)** Nucleic acid leakage of *C. acutatum*. Values are the averages of the replicates for all the analyses. Error bars are ± SD of the means. In some cases the error bar is obscured by the symbol. Columns with different letters at each time point indicate significant differences according to Duncan’s multiple range tests at *p* < 0.05.

The results of staining *C. acutatum* mycelia and spores with propidium iodide were presented in **Figures 3D–G**. The cells are intact, which cannot be stained by propidium iodide. The mycelia treated without CEO could not be stained by propidium iodide (**Figure 3E-1**), and the cell structure was integral and clear (**Figure 3D-1**). The propidium iodide penetrated mycelia treated with CEO after 3 and 6 days (**Figures 3E-2,E-3**), and the cell structure was indistinct (**Figures 3D-2,D-3**). The damage of PM of mycelia was positively correlated with treatment time of CEO. The changes of spores treated with CEO were similar to that of mycelia (**Figure 3F,G**). The propidium iodide penetrated spores treated with CEO, showing that the membrane integrity has been compromised. In summary, the permeation to propidium iodide indicated that the CEO was responsible for a fungicidal effect, resulting in extensive damage to the plasmatic membrane between mycelia and spores (**Figures 3D–G**). MI of *C. acutatum* spores declined obviously with the increase of incubation time on PDA containing MFC of CEO (**Figure 4A**). However, MI stayed at a relatively high level for spores incubated in PDA without CEO (**Figure 4A**). Moreover, results showed that the damage of the plasmatic membrane of spores was markedly more severe than the membrane of mycelia, which were in accordance with the above mentioned result that the spore germination was more sensitive to CEO treatment than that of mycelial growth (**Figures 3D–G**).

### Effects of CEO on Ergosterol Content, and Intracellular Protein, Sugar, and Nucleic Acid Leakage

**Figure 4B** showed the effects of different concentrations of CEO on the ergosterol content of the PM in *C. acutatum* compared with the control. Results indicated that the total ergosterol content was reduced with the increasing of CEO concentrations. The production of ergosterol decreased at the CEO concentrations of 0, 0.075, 0.100, 0.125, 0.150, and 0.175 μL/mL, presenting a value of 0.5885, 0.4954, 0.3750, 0.2514, 0.1412, and 0.0863%, respectively. The cells treated with CEO showed inhibition rate of 15.81, 36.28, 57.28, 76.01, and 85.34% to ergosterol compared with the control, respectively. Further, the exposure of *C. acutatum* to different concentrations of CEO caused various levels of extracellular conductivity. The extracellular conductivity in *C. acutatum* suspension was increased with exposure time and the concentrations of CEO (**Figure 4C**). All CEO treated *C. acutatum* showed higher electric conductivity than the control. Moreover, the electric conductivity increased rapidly in respond to increasing levels of CEO during the first 4 days (**Figure 4C**). After that, the growth tended to slow down. After 4 days, the extracellular conductivity of suspensions with CEO (512 μS/cm and 526 μS/cm) remained at the same level, but the values CEO treated cells were significantly higher (*p* < 0.05) than the control (211 μS/cm and 223 μS/cm) (**Figure 4C**). Additionally, the leakage of protein, sugar, and nucleic acid could be considered as important indicators of CM damage. *C. acutatum* cells were treated with the MFC of CEO, and the amounts of released protein, sugar, and nucleic acid were investigated. The results showed that *C. acutatum* cells treated with CEO accumulated more protein, sugar, and nucleic acid than the untreated group (**Figures 4D–F**). In this study, protein, sugar, and nucleic acid leakages can be detected in time-dependent tests. In time-dependent killing, protein, sugar and nucleic acid leakages were initially sluggish, and the leakages increased with treatment duration (**Figures 4D–F**). At the first 4 days, proteins and sugar leaked markedly in treatment, and the leakage increased slowly after 4 days (**Figures 4D,E**). However, the leakage of protein and sugar stayed at a very low level for control (**Figures 4D,E**). Hence, the leakages of treatment groups were significantly higher than that of control. At the first 4 days, the absorbance value for nucleic acid (OD 260 nm) of *C. acutatum* increased significantly (*p* < 0.05) from 0.018 to 0.048 (control), and from 0.013 to 1.269 (CEO treatment group), respectively (**Figure 4F**). After 4 days, the value of OD 260 nm was followed by a steady state, which clearly indicated that the CM integrity of *C. acutatum* had been compromised after exposure to CEO, which could consequently lead to cell death.

## Discussion

The isolated pathogen was inoculated in the surface-sterilized and healthy kiwifruit. The results showed that the sample was attacked after inoculating 24 h at 25°C because of brown dots on the surface of fruit. Then the lesion rapidly expanded and showed drying shrinkage and depression on the 5th day. The color of pericarp turned to dim and pink, and then sticky particulates on pathogenic sites, which were the conidial heaps. Finally, as the decay spread out, the whole of fruit was brown rot and even dried. According to the morphological and molecular identification, the pathogen from ‘Hongyang’ kiwifruit was further determined as *C. acutatum.* Previous studies found that *C. musae* occurring anthracnose could cause the development of black circular/ lenticular spots during ripening in banana (Barrera-Necha et al., 2008). So far, to best of our knowledge, there were no reports on *C. acutatum* from ‘Hongyang’ kiwifruit.

Essential oil showed a superior antimicrobial activity that the growth of tested organisms was inhibited more efficiently by gaseous contact than by solution contact (Inouye et al., 2003; Oonmetta-Aree et al., 2006). Therefore, in this study, the antifungal activity of CEO was carried out with its volatile substances, and the antifungal effect was good. The results showed that CEO exhibited antifungal activity against *C. acutatum* as a volatile *in vitro*. Both MIC and MFC of CEO were 0.200 μL/mL. These results confirmed other findings on antifungal activity of CEO against several fungal pathogens including *Aspergillus flavus*, *Penicillium expansum*, *Zygosaccharomyces rouxii* and *Zygosaccharomyces bailii in vitro* (Manso et al., 2015; Ribeiro-Santos et al., 2017; Ribes et al., 2017). CEO had a strong antifungal effect against *Aspergillus flavus*, with a MIC of 0.05–0.10 mg/mL, and a MFC of 0.05–0.20 mg/mL (Manso et al., 2013). The MIC of CEO against *Aspergillus flavus* strains and *Aspergillus oryzae* were 0.125 μL/mL and 0.250 μL/mL, respectively (Kocevski et al., 2013). CEO could effectively inhibit the growth of *Botryosphaeria parva* in the dilution method, and both MIC and MFC of CEO were 0.078 μL/mL (He et al., 2015). Volatiles of CEO obviously affected appressorium formation, while in control treatment germinal spores formed one or more appressoria. Results showed that inhibitory activity of CEO was significantly correlated with the concentration of CEO. In present study, the spore germination was more sensitive to CEO treatment than mycelial growth, which was in accordance with previous studies (Duduk et al., 2015). CEO had a fungistatic effect against mycelial growth of *C. acutatum* isolated from strawberry anthracnose at 0.667 μL/mL. Meanwhile, CEO completely prevented the spore germination and appressorium formation at 0.00153 μL/mL (Duduk et al., 2015). In addition, CEO strengthens its merits as a post-harvest fungicide against food-borne pathogens. Moreover, the MIC and MFC of CEO against *Colletotrichum* sp. were lower than those of some earlier reported EO viz., *Thymus vulgaris* L. (Zambonelli et al., 1996), clove [*Syzygium aromaticum* (L.)] (Ranasinghe et al., 2002) and tea tree oil (Szczerbanik et al., 2007). These results indicated that CEO had high potential of economic exploitation.

The potential mechanisms underlying the antifungal activity of plant essential oils are not fully understood, but a number of possible mechanisms have been proposed. The results of TEM indicated that the CEO destroyed not only the CW, but also the PM, by interacting with the structures of cytoplasmic organelles in comparison with untreated samples. The degree of damage had dose-effect relationship, and that destructiveness of 2MFC was stronger than MFC. The CEO showed antifungal activity against *C. acutatum* causing cellular damages and irreversible morphological changes. Previous studies revealed morphological alterations in *Aspergillus flavus* by TEM observations (Nogueira et al., 2010). Results showed that a marked disruption of membranes of major organelles such as nuclei, mitochondria and endoplasmic reticulum, indicated that *Ageratum conyzoides* EO passed not only through the CW, but also through the PM and then interacted with membranous structures of the cytoplasmic organelles. Studies also reported irreversible damage to CW, CM and organelles of *Aspergillus flavus* by *Cinnamomum jensenianum* essential oil (CJEO) (Tian et al., 2012). In the CJEO-treated hyphae, the fibrillar layers had gradually lost their integrity, becoming thinner, and eventually failing to deposit on the CW. In addition, the mitochondria suffered a severe disruption of the internal structure with complete lysis. Indeed, studies demonstrated that the TTO could destruct for organelles of *Botrytis cinerea* by TEM observation (Yu et al., 2015). The PM appears to be the main target of the EO according to TEM data. Furthermore, FMS observation showed that the propidium iodide could permeate CM into intracellular cytoplasm while cells were destroyed. Then CEO was responsible for a fungicidal effect, resulting in extensive lesion to the plasmatic membrane, either from a direct effect or as a secondary result of metabolic impairment. Results showed that CEO could be used as a fungicide to damage membrane integrity.

In order to confirm the CEO targets in the PM, the amount of ergosterol was assessed. Ergosterol is the major sterol component of the fungal CM, helping to maintain cell function and integrity (Vale-Silva et al., 2010). It is a sterol with a CM specific of fungi and microalgae with the advantage of indicating only viable biomass, since it is quickly degraded after the cell’s death (Gutarowska and Zakowska, 2009). The correlation between ergosterol and biomass of several fungal species has been confirmed (Khan et al., 2010; Silva et al., 2010). Previous studies suggested that the PM was the main target of EOs against fungi, and that the oil caused dose-dependent reduction in ergosterol quantity (Tian et al., 2012; Yu et al., 2015). Our results supported a model in which cellular membranes were the primary targets for CEO with different concentrations.

The lipophilicity of EOs enable them to preferentially part from an aqueous phase into membrane structures of the fungi, resulting in expansion of membrane, and then increase of membrane fluidity and permeability, disturbance of membrane-embedded proteins and soluble sugars, and other cellular contents. The electric conductivity was examined to express the changes of CM permeability. Our results showed that the extracellular conductivity rose with the increasing of CEO concentration and treatment time. The results clearly indicated that there was a leakage of electrolytes due to the disruption of cell permeability caused by CEO. Excessive electrolyte loss would cause the death of *C. acutatum.* This slight increase for control might be due to regular fungal cytolysis and death, just as Diao et al. (2014) and Zhang et al. (2016). The integrity of the cytoplasmic membrane is a critical factor to fungal growth. Analyzing the leakage of cell constituents could therefore give further insight into the mechanism of antifungal action. The ability of CEO to disturb the integrity of the PM of fungal cells was also assessed by measuring the protein, sugar, and nucleic acid released in cell suspensions. Results showed that the leakage increased with the extension of treatment time.

The antifungal property of EO may be contributed to its major components. 1, 8-cineole (56%) was the major component in *Callistemon lanceolatus* (Sm.). Sweet essential oil, which showed great inhibitor effect against fungi (Shukla et al., 2012). (*E*)-cinnamaldehyde was found as the major component in cinnamon leaf volatile oil, which possessed crucial inhibitory activity (Singh et al., 2007). The compositions of CEO are greatly influenced by the species, part of plant used, geographic origin, time of harvest, stage of development, age of plants and extraction method (Lee et al., 2005). Twenty components in CEO extracted from bark was identified, and twenty-one components of CEO extracted from leaf was identified. The major components of bark EO are methyl cinnamate (81.87%), linalool (3.90%) and α-pinene (2.41%). The major components of leaf volatile oil are linalool (67.60%), methyl cinnamate (17.32%) and α-pinene (2.74%) (Malsawmtluangi et al., 2016). Previous studies have validated that the major components of CEO are *trans*-cinnamaldehyde or cinnamaldehyde (Goñi et al., 2009), which were consistent with our results (**Table 1**). Furthermore, the content of *trans-*cinnamaldehyde (86.16%) in our study was much higher than that of previous studies (Lv et al., 2011; Li et al., 2013). It remains to be further defined whether major components are the main antifungal composition.

## Conclusion

Results demonstrated that the major pathogen from ‘Hongyang’ kiwifruit causing to anthracnose was *C. acutatum.* The inhibition of CEO against *C. acutatum* attributed to the reducing of fungal growth and spore germination. CEO could penetrate CW, and pass through the PM, and then interact with the membranous structures of cytoplasmic organelles. Moreover, the major components of CEO such as cinnamaldehyde could be used as a natural fungistat. The antifungal mechanism of cinnamaldehyde requires further investigations.

## Author Contributions

JH, WQ, and DW designed the study. JH, HL, QH, XL, HW, YW, and JY performed the experiments. JH drafted the work. JH, DW, QZ, HC, and HD wrote and revised the manuscript. JH, WQ, and DW revised the final version to be published.

## Conflict of Interest Statement

The authors declare that the research was conducted in the absence of any commercial or financial relationships that could be construed as a potential conflict of interest.

## Footnotes

1 http://blast.ncbi.nlm.nih.gov
